# Supplementary material for: Behavioral fingerprints predict insecticide and anthelmintic mode of action
Source: Mol Syst Biol. 2021 May 25;17(5):e10267. doi: 10.15252/msb.202110267 (PMC8144879; doi:10.15252/msb.202110267)
Supplement: Supplementary file 1 — Expanded View Figures PDF [file MSB-17-e10267-s003.pdf]

Expanded View Figures

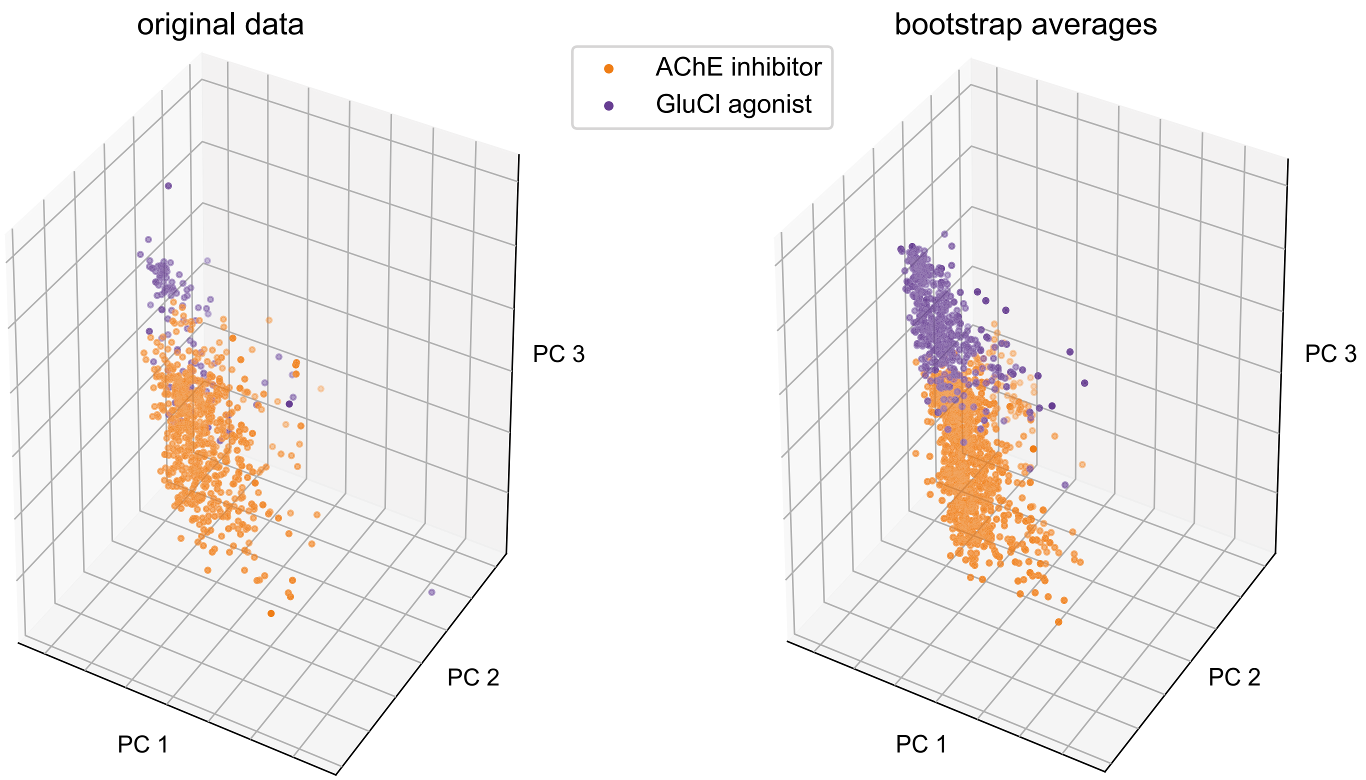

Figure EV1. The smoothing and balancing procedure using bootstrapped averages reduces the effect of outliers and balances the classes increasing classification accuracy.

One of the sparsely populated classes (GluCl agonists with 5 compounds) and the most well-populated class (AChE inhibitors with 10 compounds) are shown before and after the smoothing and balancing procedure in the 3 top PCA components.

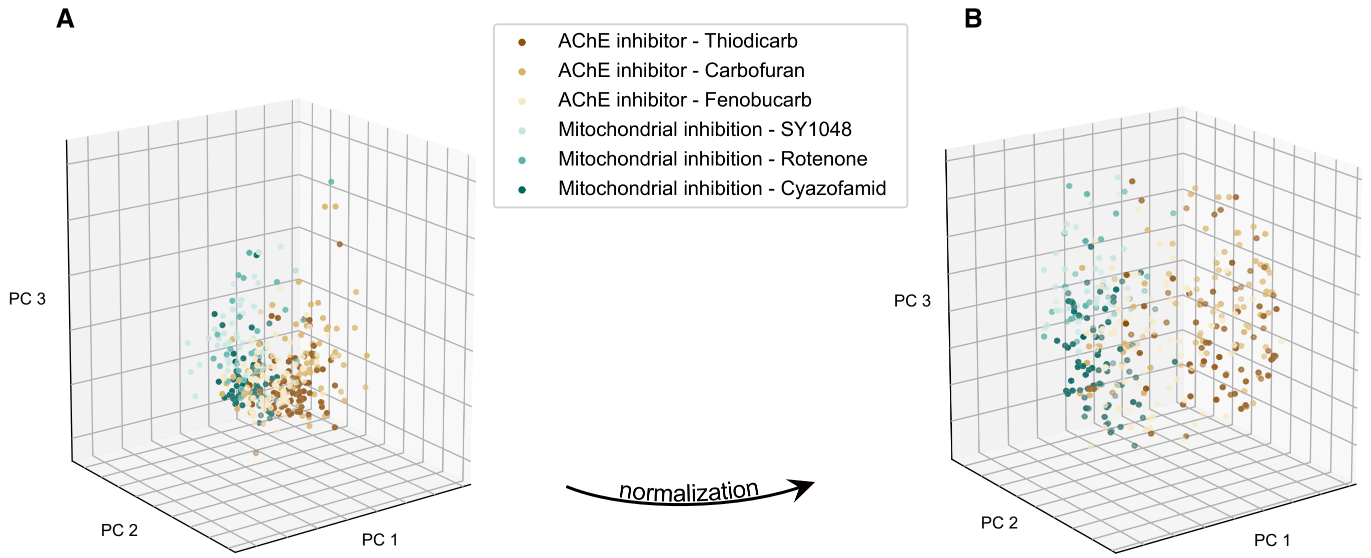

Figure EV2. Illustration of the effect of normalization with real data.

The normalization of samples to unit L2 norm brings compounds with different potencies closer together and helps separate the classes in the phenotypic space.

A PCA of the data from 2 different classes with 3 compounds each before normalization (data simply standardized).

B PCA of the normalized data.

 © 2021 The Authors

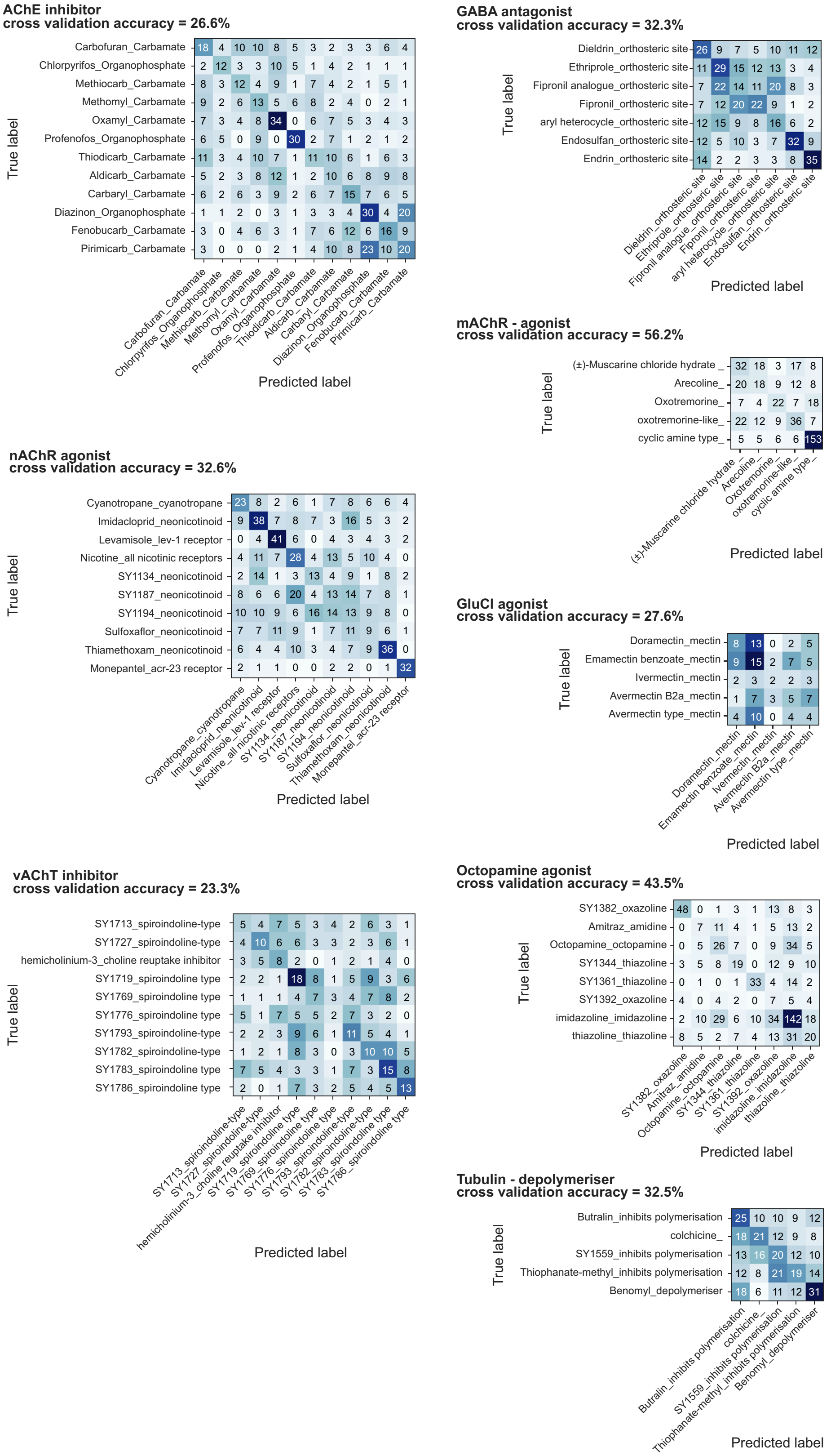

Figure EV3. Confusion matrices showing cross-validation performance of a classifier trained to distinguish compounds of the same class from each other.

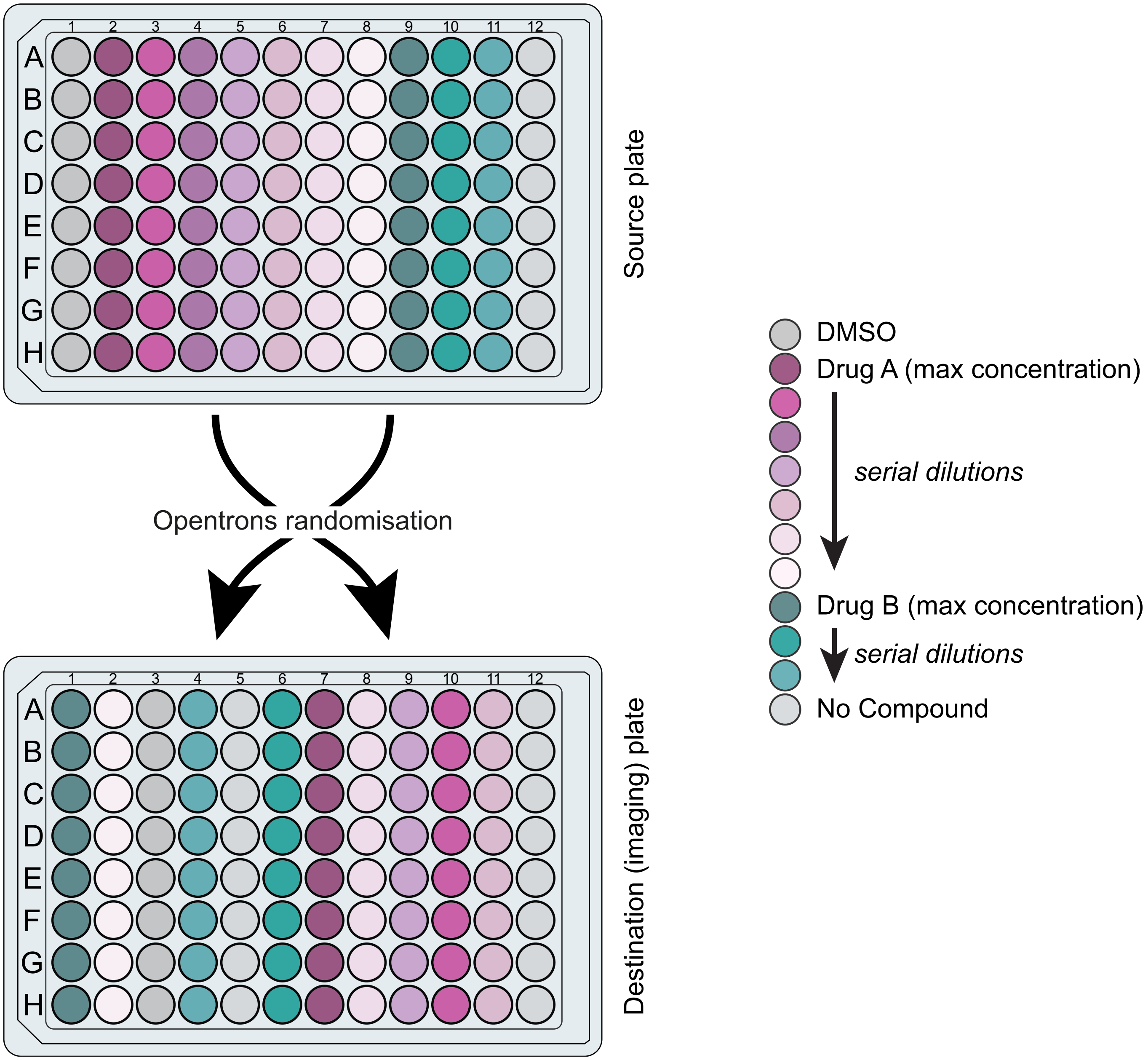

Figure EV4. Randomization of columns using liquid handling robot.

To minimize any position-induced bias, we use an Opentrons liquid handling robot to randomly shuffle the position of the compounds in the imaging plates. We programmed the robot to keep a record of the randomized shuffle and use this log to create the correct well-compound mapping.

 © 2021 The Authors

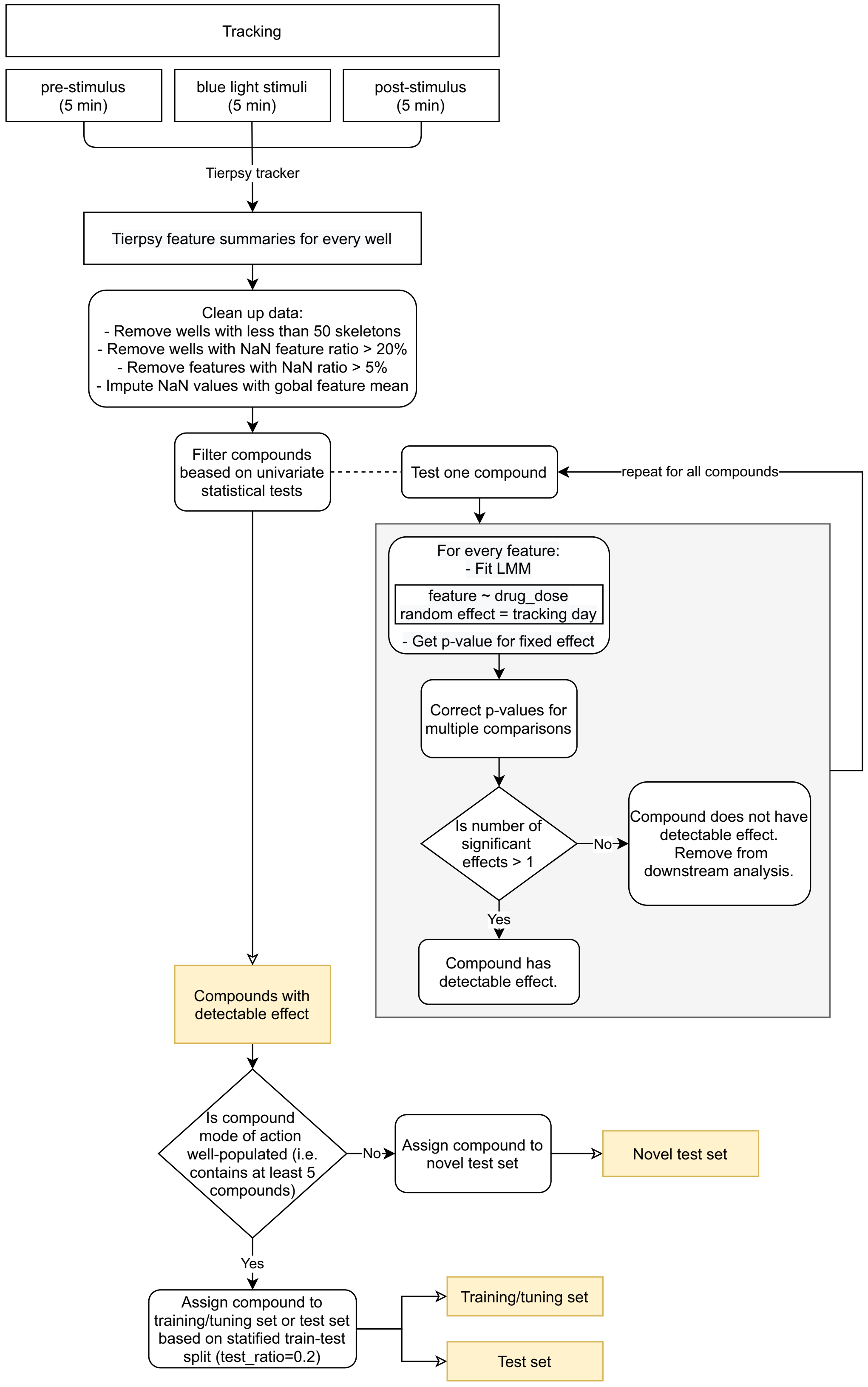

Figure EV5. Flow chart showing the data pre-processing steps.

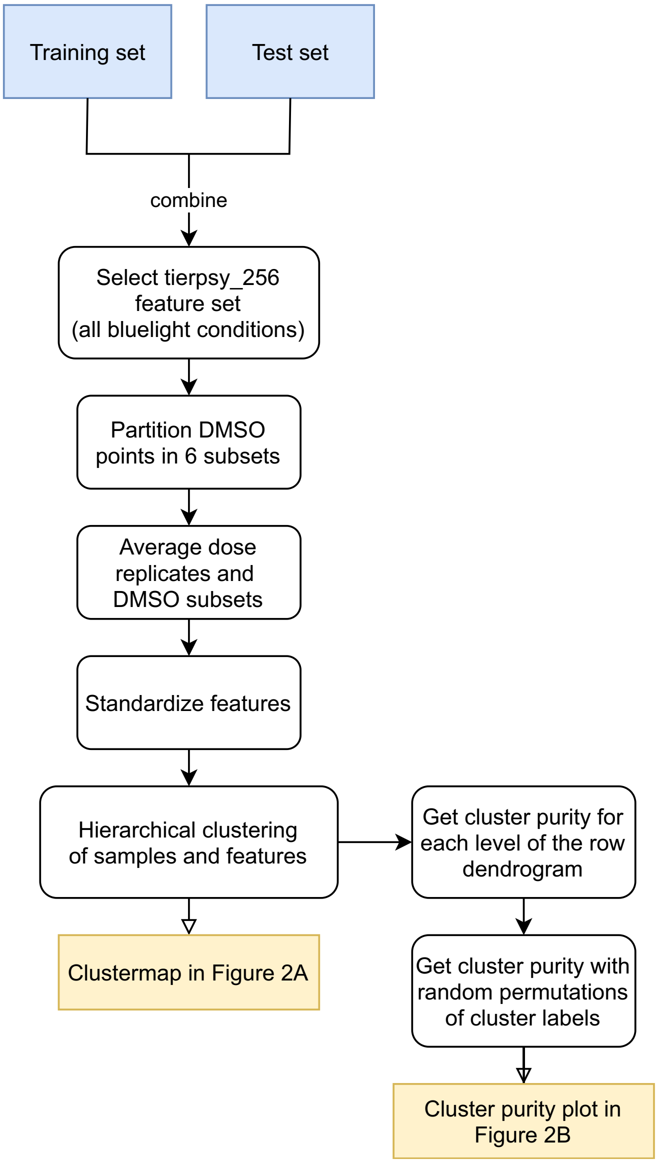

Figure EV6. Flow chart showing the pipeline for the clustering of average drug doses.

© 2021 The Authors

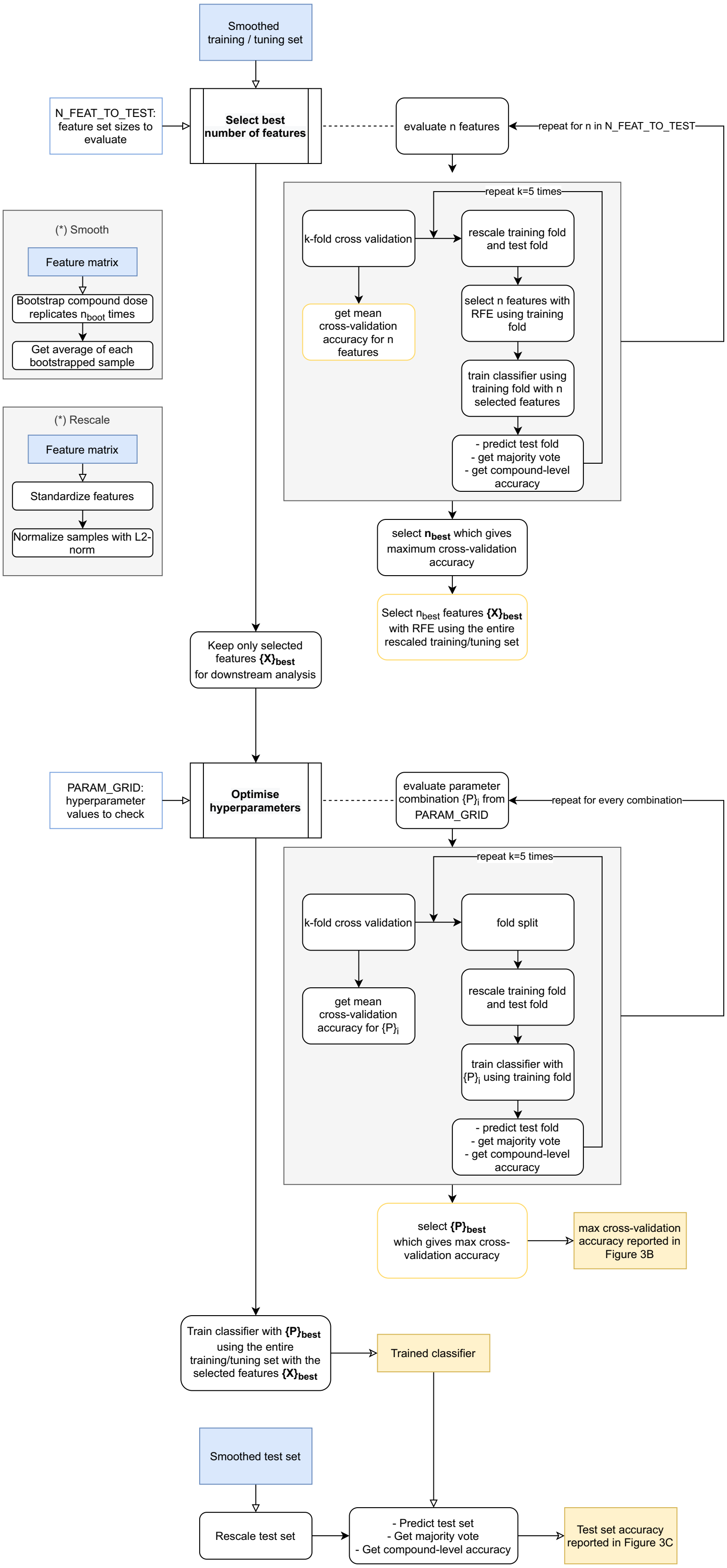

Figure EV7. Flow chart showing the steps followed for the tuning and training of the classifier using the training dataset and the prediction of the mode of action in the test set.

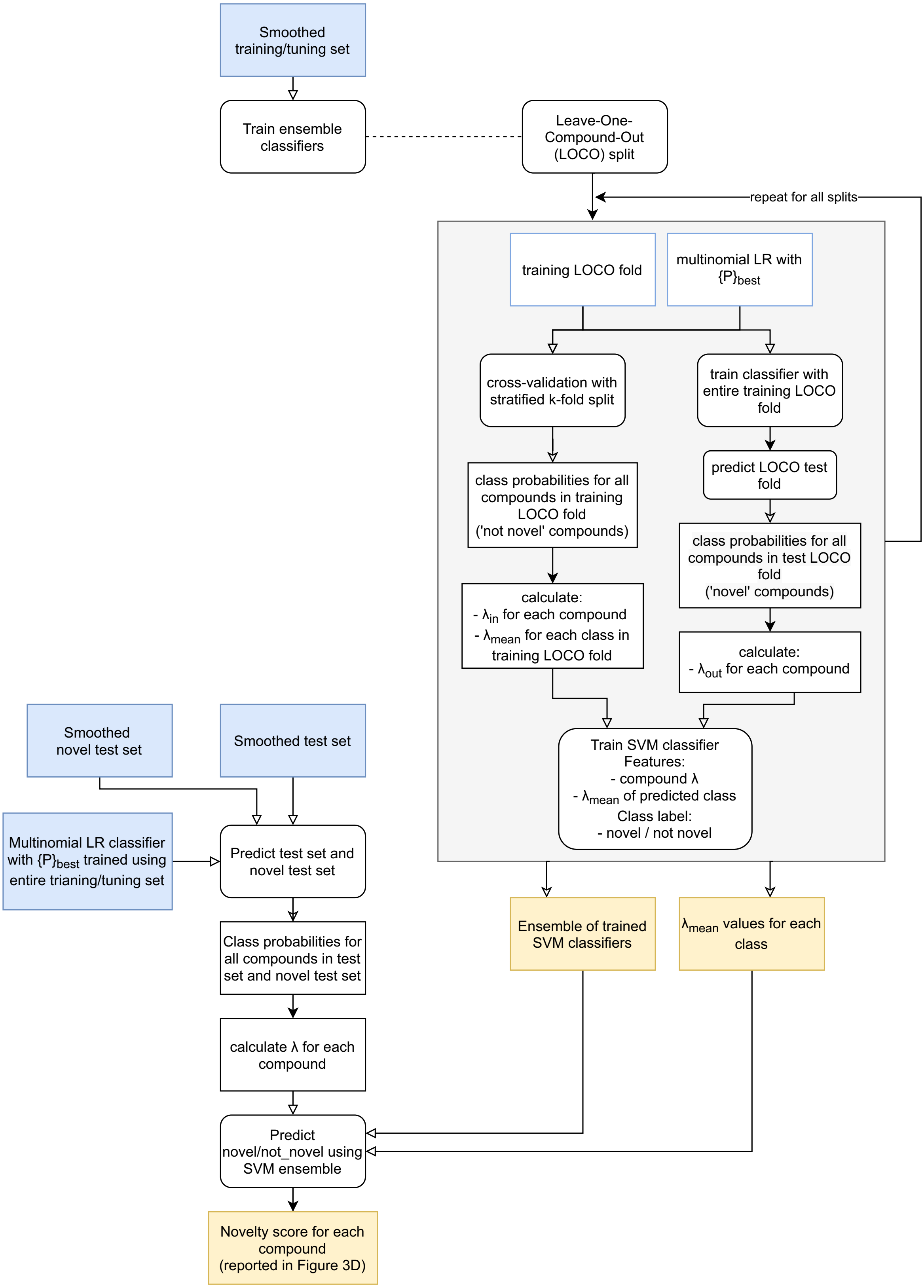

Figure EV8. Flow chart showing the steps of the novelty detection method.

© 2021 The Authors
